# Regional Social Development Gap and Regional Coordinated Development Based on Mixed-Methods Research: Evidence From China

**DOI:** 10.3389/fpsyg.2022.927011

**Published:** 2022-07-01

**Authors:** Weiwei Liu, Zhiwei Liu, Lian Wang, Haiming Liu, Yan Wang

**Affiliations:** ^1^School of Management, Zhejiang University of Technology, Hangzhou, China; ^2^Wenzhou Polytechnic, Wenzhou, China; ^3^School of Education, Central China Normal University, Wuhan, China; ^4^Student Affairs Office, Zhejiang University of Science and Technology, Hangzhou, China

**Keywords:** regional social development gap, regional coordinated development, 35 large and medium cities, comprehensive evaluation, mixed-methods research

## Abstract

Due to the continuous acceleration of the global urbanization process, the unbalanced development of regional cities has become an unavoidable reality under the rapid economic and social development of China. Unbalanced social development will affect coordinated and sustainable economic development, regional ethnic unity, and political and social stability. This research uses data from the 2011–2015 period, 2016–2020 period, and various special development plans of 35 large and medium cities, combines qualitative analysis and quantitative analysis, establishes a comprehensive evaluation model, and conducts cluster analysis, using standard deviation. The coefficient of variation aims to measure and study whether the gap in China’s regional social development has continued to widen over the past decade. This study found that: (1) From the overall national perspective, there are obvious differences in the level of social development in the development plans of 35 large and medium-sized cities. The social development level of each large and medium-sized city has been improved to a certain extent, and the social development gap between cities has also been reduced to a certain extent. (2) From the 2011–2015 period to the 2016–2020 period, the social development gap between the three regions of my country’s eastern, central, and western regions has declined. (3) The trend of social development disparity within the three major regions of the eastern, central, and western regions is not the same. The internal social development gap in the eastern region shows a downward trend, while the internal social development gap in the central and western regions shows an upward trend. This study provides a valuable reference for rapidly urbanizing developing countries in the process of globalization.

## Introduction

It is one of China’s basic national conditions that China is a developing country with vast territory, a large population, and uneven development. This is also the objective basis for the Chinese government to formulate effective economic and social development plans and policies. Since the reform and opening-up in 1978, China’s economic and social development has achieved remarkable achievements, creating the “Chinese Miracle” (Chen et al., 2016). With the rapid development of China’s economy and society, the regional spatial distribution of China’s economic activities has undergone great changes. The regional concentration of economic activities has become higher and higher. In the early phase of reform and opening-up, China’s “Three-Step” strategy had encouraged unbalanced regional development (Fan, 1997). Priority development in eastern China (Feng and Humphreys, 2012) accelerated economic development but also created imbalances in regional sustainable development. Factors and economic activities are concentrated in the eastern coastal cities, and the regional spatial structure is unbalanced, resulting in a huge gap in economic development between regions.

The question of whether regional development gaps in socialist countries have decreased and whether the economic reforms undertaken by the Chinese government since 1978 have increased regional development gaps has attracted considerable attention (Koropeckyj, 1972; Fuchs and Demko, 1979; Forbes and Thrift, 1987; Ozornoy, 1991; Smith, 1996). Some believe that unbalanced regional development is the geographical manifestation of the internal structural contradictions of capital in the process of urban development, and is an inevitable process in regional development (Smith, 1991; Hui et al., 2016). With the rapid development of globalization, urbanization, and regional economic integration, unbalanced and insufficient regional economic development has become an important aspect of spatial organization and a global problem. As a shared product of urbanization development and regional economic development, there are often close economic and social ties within the spatial scope of China’s urban land (Wu et al., 2015). The development of economic globalization has affected the transformation and promotion of city importance to a certain extent (Shen, 2007; Wu and Zhang, 2007). Cities are often considered one of the means of national regionalization management (Lin, 2014; Wu, 2017). Especially due to the unbalanced development and high environmental costs in the process of economic growth, the coordinated development of regions within urban agglomerations has become more pronounced since the 1990s. A large number of theoretical and empirical studies have shown that regional coordinated development is key to alleviating the disadvantageous factors of globalization and sustainable development (Dabson, 2010; Hirschi, 2010; Kim et al., 2011; Mukul, 2012; Bruce, 2014). Although the research on the regional economic and social development level gap is a well-studied topic, scholars mainly focus on the status quo and changing trends or analysis of the reasons for the formation and evolution of regional social development gaps. However, due to different research angles and different analysis methods used, the final results differ greatly. Entering the 21st century, China’s domestic and foreign policy environment has undergone great changes. Under the background of today’s new economic and social development, China’s economic and social development must also have taken on new characteristics.

Development planning is essentially a major administrative decision. Government decision-making is the primary link for the government to implement effective management (Zhang P. et al., 2017; Chen et al., 2019; Ma et al., 2019). China’s development plan (also known as the national economic and social development plan, referred to as the “five-year plan”) started from the planned economy period in 1953. It was initially issued as a command-and-order plan, and then gradually developed into a strategic, overall, programmatic and guiding plan. The content of development planning is also updated with the continuous development of the times, including economic, social, environmental, ecological, and other fields (Xu, 2014; Shi et al., 2014, 2018). At the same time, the spatial differences in China’s development plans are also becoming more and more obvious. According to the basic development situation of different urban areas, the differences in planning content between different cities are becoming more and more obvious. Nowadays it has become an essential time and spatial guiding strategy (Hsing, 2006; Wang et al., 2017; Li et al., 2018; Zeng et al., 2018). To a certain extent, development planning has become an important means for the Chinese government to guide and manage social development, which plays an increasingly important role in achieving national strategic goals.

The new urbanization strategy is a long-term development strategy that China will implement in the future. City-level cities are the main subject of the continuous advancement in the new urbanization process, and the most direct subject of public resource allocation, such as land resources, water resources, etc. (Zhao, 2010; Li et al., 2013). Secondly, the city is the “operator” of new urbanization, the main body of monitoring various economic operation indicators, policy discretion, and the implementation of social policies, which can form a “face-to-face” relationship with the public, and directly perceive economic and social development and changes with in-time responses (Zhu et al., 2019). Therefore, many reforming experiments of government policies are usually carried out at this level. At present, China is in the stage of rapid urbanization, and the scale and number of cities are in rapid growth. According to the division of administrative levels of cities in the *China Urban Statistical Yearbook*, Chinese cities can be divided into municipalities (4), sub-provincial cities (15), prefecture-level cities (260), and county-level cities (386). According to the *City Planning Law of the People’s Republic of China*, cities can be divided into super cities (15), megacities (30), large cities (64), medium cities (225), and small cities (326) according to their size. According to the geographic location of cities, cities can be divided into eastern cities (287), central cities (247), and western cities (126).

The compilation of development planning is its “starter” in the process of regional development. The changing trend of the regional development gap has a profound theoretical value from the compilation content of the development plan. After consultation and discussion with leaders of government departments and relevant experts and scholars, based on the four principles of representativeness, authenticity, difference, and feasibility of the selection of research objects, and per previous relevant research literature, this study decided to select 35 large and medium-sized cities in China as the analysis objects to study the regional development gap in China in the past 10 years. The research results can provide valuable references for rapidly urbanizing developing countries in the process of globalization.

## Materials and Methods

### Literature Review

Unbalanced regional development is one of the prominent problems in China’s economic and social development, which affects the sustainable development of society and economy, and is also a common problem in the development of countries around the world (Brenner, 1999; Acuto, 2018; Zhang and Wang, 2018). It has been a subject of intense debate among various schools, especially about its convergence and divergence. The idea of long-term convergence is consistent with the growth pole theory and inverted-U theory, as proposed by Alonso (1980), among which unbalanced social development rises in the early stages of development and declines as the economy matures. However, Smith (1980) regards the persistence of regional social development gaps as a necessary premise and inevitable result of capitalism. Out of worry about globalization and liberalization, globalization since the late 1980s has once again provoked debates about widening disparities in regional social development. Barro and Sala-i-Martin (1991) and others further divide the concept of convergence into σ convergence (thinking that overall dispersion, in the long run, goes down) and β convergence (indicating that poor areas grow faster than rich areas). Fujita et al. (1999) argue that the new economic geography places the benefits of increased scale and agglomeration at the center of regional development. Storper (2018) argue that due to the influence of various factors such as geographical location, climatic conditions, historical reasons, and policy factors, it is difficult to maintain a balanced development between regions, and the development gap between regions will gradually widen. Li (2012) studies regional differences from the perspective of the Human Development Index and finds that the overall difference in HDI is narrowing. Kanbur and Zhang (1999) establishes a unified empirical framework to describe the relative contribution of China’s inland coastal regional development imbalance to the overall regional development gap in the 1980s and 1990s.

Early research on China’s regional development imbalance mainly focused on measuring regional development imbalance (Tsui, 1991; Rozelle, 1994; Kanbur and Zhang, 1999). After that, scholars conducted an in-depth decomposition and discussion of the factors affecting China’s regional imbalance (Wan, 2001; Wan and Zhou, 2005). Recently, attention has been paid to analyzing imbalances and poverty at the taxonomic level of counties, villages, households, and even individuals (Meng et al., 2017; Zhang L. X. et al., 2017). In fact, the proper allocation of resources to regions has become an important part of China’s national planning. In 1953, the Chinese government implemented its first Five-year plan (1953–1957), which planned to transfer resources to inland areas through the government’s centralized fiscal and investment system. Under this strong intervention of government policies, the degree of regional imbalance in China had decreased (Paine, 1981; Wu, 1987; Pannell, 1988). Since the reform and opening-up in 1978, China’s regional development policy has shifted from self-reliance to reform and opening up. Since the Chinese government encouraged coastal areas to “get rich quick,” China’s eastern coastal areas have experienced rapid socio-economic development ever since, while some inland areas have lagged far behind. Researchers argued whether China’s regional development gap has widened. Some scholars believe that the Chinese government’s economic reforms, especially the coastal development strategy and opening-up policy, stimulated the economic and social development of coastal provinces and exacerbated regional imbalances. Researchers believe that the Chinese government should devote more resources to developing poorer inland areas to reduce regional imbalances, and they advocate shifting policy focus from the coast to the interior (Brown, 1991; Taylor, 1993; Agnew and Corbridge, 1995; Hu and Kang, 1995). However, not all scholars accept the view that the regional development gap has widened since China’s reform and opening-up in 1978. Instead, they argue that regional imbalances have declined since 1978, mainly due to diffusion, inter-regional resource transfers, and rural industrialization. Economic reforms have not exacerbated regional development imbalances but have brought economic prosperity and opportunity to all. They advocate continuing coastal-oriented regional development policies (Chen and Fleisher, 1996; Jian et al., 1996). Other scholars believe that due to the influence of the growth pole and the inverted-U theory, temporary expansion of regional development gaps is inevitable. China should pursue the goal of economic efficiency, and the diffusion effect in the process of regional development will promote the development of poor areas, which ultimately reduces regional imbalances (Liu et al., 1994; Yang, 1994).

#### Recent Relevant Literature

In recent years, the research focus has been shifted from the original regional economic growth to environmental regulation and green development, paying more attention to sustainable development research in the process of regional development. Wu et al. (2019) used the entropy method to comprehensively evaluate the level of urbanization in China and used a dynamic threshold panel model to study the relationship between energy consumption and different stages of comprehensive urbanization. The empirical results show a non-linear relationship between energy consumption and urbanization, and the energy consumption is conducive to promoting China’s current urbanization process. However, with the increase of energy consumption intensity and scale of energy consumption, the positive effect weakens, and with the improvement of energy consumption structure, the positive effect increases. Wu et al. (2020) used panel data of 30 provinces in China from 2005 to 2016 to examine the potential non-linearity between environmental regulation and China’s GTFEE under different environmental decentralization conditions through a spatial Durbin model and a dynamic threshold panel model. The results show a significant U-shaped relationship between environmental regulation and GTFEE in China. With further expansion of environmental decentralization, local governments’ autonomous choices for pollution control have been enhanced. The improvement of environmental decentralization will lead to the negative moderating effect of environmental regulation on GTFEE. Furthermore, regression results from dynamic threshold models suggest that environmental decentralization increases the negative impact of environmental regulation on GTFEE. The nonlinear impact of environmental regulation on GTFEE depends on the specific type of environmental decentralization. A higher degree of environmental decentralization will lead to a stronger binding effect of environmental regulation on GTFEE. Ren et al. (2022) discussed the impact of OFDI and institutional quality on GTFEE using a spatial Durbin model based on panel data of 30 provinces in China from 2006 to 2017. The results of the study showed that the inter-provincial GTFEE had a significant spatial correlation. OFDI can improve not only the local GTFEE but also the GTFEE of neighboring regions. OFDI can increase the GTFEE of the home country by increasing technological innovation, upgrading the industrial structure, and mitigating capital mismatches. The results of the threshold effect suggest that the nonlinear effect of OFDI on GTFEE depends on institutional quality. Higher levels of corruption lead to a diminished role of OFDI in promoting GTFEE. The level of marketization and the improvement of intellectual property protection can increase the positive impact of OFDI on GTFEE. Wu et al. (2021), based on the panel data of 30 provinces in China from 2006 to 2017 and 196 cities from 2011 to 2018, used OLS, spatial Durbin model, threshold model, mediation effect model, and DID model to study the impact of Internet development on the impact of energy-saving and emission reduction. The empirical results show that the development of the Internet can improve the efficiency of energy conservation and emission reduction through technological progress, energy structure, human capital, and openness. At the same time, the impact of Internet development on energy conservation and emission reduction efficiency in adjacent areas also has a significant positive spatial spillover effect, which still exists under the spatial weight matrix of different distances. The impact of Internet development on energy conservation and emission reduction efficiency is related to technological progress, energy structure, human capital, and openness are nonlinear at different levels. Yang et al. (2021) adopted a dynamic spatial Dobin model of the economic-geographical weight matrix, taking 269 prefecture-level cities in China from 2004 to 2018 as the research object, to analyze the direct and regulatory effects of fiscal decentralization and urban expansion on air pollution. The results show that air pollution has a significant delay time effect and spatial spillover effect. Both fiscal decentralization and urban sprawl have had a major impact on air pollution. From the perspective of short-term effects, the coefficients of urban expansion and fiscal decentralization on the total spillover effect, direct spillover effect, and indirect spillover effect of air pollution are significantly positive, respectively. From the perspective of long-term effects, the overall spatial spillover effect of urban expansion and fiscal decentralization on air pollution is significantly negative, and the direct and indirect effects are both negative but not significant. Chai et al. (2021) used the economic growth target data from the work reports of 30 provincial governments in China from 2006 to 2017 to construct various spatial measurement methods such as the spatial Durbin model to examine the impact of economic growth targets on air pollution constraints. The research results show a significant U-shaped relationship between the constraints caused by economic growth goals and air pollution, and PM2.5 in each province in China exhibits significant positive spatial spillover effects and spatial agglomeration characteristics. The direct, indirect, and overall effects of air pollution are all statistically significant in a U shape.

After sorting out the existing research literature, it is found that, from the perspective of research content, most of the existing research on regional development is analyzed from a single perspective such as economic development or green development, and less research and analysis are conducted from the comprehensive perspective of social development. In terms of sources, most current research directly uses the data from the China Statistical Yearbook and lacks analysis from the start of the regional development process of government development planning. Mainly, there is a lack of research from the perspective of sub-regional and sub-urban agglomeration with only provincial and prefecture levels. Therefore, the author takes the development plans issued by 35 large and medium-sized cities in China from 2011 to 2020 as the research object to study the regional development gap in China in the past decade.

### Study Area and Data

In 1997, with the approval of the State Council, the State Development Planning Commission and the National Bureau of Statistics decided to carry out the compilation of real estate price indices in 35 large and medium-sized cities with rapid economic development and a large proportion of real estate development investment in those regions. The concept of *35 large and medium-sized cities* was proposed for the first time. The 35 large and medium-sized cities include four municipalities: Beijing, Shanghai, Tianjin, and Chongqing; 15 sub-provincial cities under separate state planning: Harbin, Changchun, Shenyang, Jinan, Nanjing, Hangzhou, Guangzhou, Wuhan, Chengdu, Xi’an, Dalian, Qingdao, Ningbo, Xiamen, Shenzhen, of which Shenzhen, Ningbo, Qingdao, Xiamen, and Dalian; 16 prefecture-level cities: Shijiazhuang, Zhengzhou, Nanchang, Taiyuan, Urumqi, Hohhot, Changsha, Guiyang, Kunming, Xining, Lanzhou, Yinchuan, Nanning, Fuzhou, Haikou, and Hefei. Among them, there are 17 cities in the eastern region, accounting for 48%; 9 cities in the central region, accounting for 26%; and 9 cities in the western region, accounting for 26% of the total. The 35 large and medium-sized cities are distributed in various provinces and geographically dispersed, covering cities in different geographical locations in each province, which meet the requirements of the study of social development differences and are highly representative. These 35 large and medium-sized cities cover the main economic core areas in China. The social and economic development of these cities is relatively mature, and the speed of social development can truly reflect the market supply and demand relationship with its changing trends. The 35 large and medium-sized cities are political center cities (provincial capital cities) or economic development center cities. They have a special status in economic development, political status, and legal formulation. They are the focus of national economic and social development, and can better meet the needs of regional difference research.

Due to the typical representation of China’s 35 large and medium-sized cities, a large number of scholars have taken the 35 large and medium-sized cities in China as research objects for Chinese urban land research (Wang and Zang, 2017; Lan et al., 2018; Wang, 2018; Zhu et al., 2019), Housing and Population Research (Wang and Zhang, 2010; Wang and Cao, 2013; Xia and Lu, 2015), Urban Development Research (Shen and Zheng, 2016; Wang and Hui, 2017; Liu et al., 2018).

After consultation and discussion with leaders of government departments and relevant experts and scholars, based on the four principles of representativeness, authenticity, difference, and feasibility of the selection of research objects, and referring to the relevant research literature in the past, this study decided to select 35 large and medium-sized Chinese cities as the research object. Specifically, the data used in this study come from the 2011–2015 annual development plans, 2016–2020 annual development plans, and corresponding year’s special development plans for social and economic development published on the official websites by governments of these 35 large and medium-sized cities.

### Methods

For a long time, empirical research on regional development gaps has been plagued by methodological problems. The ways adopted in which regional development imbalances are measured can also affect the findings. At present, the main methods to investigate the development gap between regions are Gini Coefficient, Kakwani index, and Atkinson index (Zou et al., 2006; Ngai and Pissarides, 2007; Zhao et al., 2015). However, these methods are based on statistical data (GDP) or social survey data. With the acceleration of social and economic development in China, these methods have many problems in terms of temporal resolution and accuracy. For example, the inconsistent and slow update cycle of GDP data across regions has led to questionable accuracy. Therefore, it is necessary to seek a new alternative or complementary method to measure the regional development gap.

Based on existing literature in the research area and actual development of the area, this study adopts the *National Economic and Social Development Planning Outline* officially issued by the municipal governments in China to construct a comprehensive evaluation index system for social development in 35 large and medium-sized cities in China. Through standardizing processing, the study uses the entropy weight method to determine the weight of each parameter based on the 2011–2015 annual development plan and the 2016–2020 annual development plan data and uses factor analysis to compare the levels of social development in 35 large and medium-sized cities during the phase of 2011–2015 and 2016–2020. Also, the study classifies the social development levels of 35 large and medium-sized cities by cluster analysis method, using relevant statistical indicators such as standard deviation and coefficient of variation, and discusses the trend of regional social development gap and the social development gap in eastern, central, and western regions.

#### Evaluation of the Social Development Level of 35 Large and Medium Cities

##### Data Standardization

The empirical evaluation data comes from the 2011–2015 Annual Development Plan, 2016–2020 Annual Development as well as a special Development Plan Outline from 35 large and medium-sized cities in China published on the official websites by municipal governments. After extracting the text data, the observed data values of the indicators are obtained. Then, it cannot be directly substituted into the factor analysis model. In order to make these data types comparable, their dimensional characteristics need to be eliminated. Therefore, the data used in this study was first standardized. The calculation formula for the normalization process is as follows:

Positive indicators:


(1) $$Z_{i}=\frac{X_{i}-\text{min}\,\left(X_{i}\right)}{\max\left(X_{i}\right)-\text{min}\,\left(X_{i}\right)}$$

Negative indicators:


(2) $$Z_{i}=\frac{\max\left(X_{i}\right)-X_{i}}{\max\left(X_{i}\right)-\text{min}\,\left(X_{i}\right)}$$

Among them, *Z_i_* is the value after data standardization, *X_i_* is the original data value of the i-th indicator, min (X_i_) is the minimum value of the i-th indicator’s original data value, and *max*⁡(X_i_) is the original data value of the i-th indicator. The maximum value among data values.

##### Entropy Method to Determine the Weight

In information theory, entropy is a measure of uncertainty. As the amount of information increases, the value of entropy will gradually decrease (Tang, 2015). This method can eliminate the people and uncertainty in the evaluation analysis to the greatest extent, avoid the deviation caused by subjective influence, and make the evaluation objective (Zou et al., 2006; Ngai and Pissarides, 2007; Zhao et al., 2015). The more effective information provided by the indicator, the smaller the entropy value, which indicates that the greater the degree of dispersion of the indicator, the greater the weight of its influence (McGarigal and Marks, 1995; Wang et al., 2014; Zhao et al., 2015). Therefore, this study uses the calculation of entropy to determine the weight of each parameter in the evaluation index of the production-life-ecological space function. The detailed steps are as follows:


(3) $$\mathrm{Proportioncalculation}:P_{i}=\frac{X_{i}}{\sum_{i=1}^{n}X_{i}}$$

(4) $$\text{Entropycalculation}e_{i}=-k\sum_{i=1}^{n}P_{i}\ln\left(P_{i}\right)$$

(5) $$\mathrm{Weightcalculation}:W_{i}=\frac{1-e_{i}}{\sum_{i=1}^{n}\left(1-e_{i}\right)}$$

(6) $$\text{Comprehensivescorecalculation}S_{i}=\sum_{i=1}^{n}W_{i}\times P_{i}$$

Among them, *P_i_* is the normalized value of the original data, *X_i_* is the original data value, e_i_ is the entropy value of the index, W_i_ is the weight value of each index, S_i_ is the comprehensive development level score of each city, k=*ln*⁡(n) > 0, satisfies *e*_*i*_≥0.

##### Evaluation Index System of Social Development Level of 35 Large and Medium Cities

This research analyzes the basis for the construction of the social development evaluation index system based on the basic goals of the social development and construction proposed in the 2011–2015 and 2016–2020 development plan outlines, keeping to the principles of uniformity, accessibility, functionality, and completeness to the construction of the statistical indicator system. Drawing on some representative literature research results (Wang and Zang, 2017; An, 2018; Bei, 2018; Wei and Li, 2018; Shi and Li, 2019), the study constructs a social development evaluation index system from the employment, social security, education, medical and health, population and travel, pollution control, and ecological greening in these 35 large and medium-sized cities. Finally, it forms the evaluation index system of 35 large and medium cities shown in Table 1.

**TABLE 1:** Evaluation index system of social development level of 35 large and medium cities.

Target level	First-level indicator level	Secondary indicator lever	Indicator code
Evaluation Index System of Social Development Level of China’s 35 Large and Medium Cities (A)	Employment (A1)	The growth rate of per capita disposable income of residents (%)	A11
		Accumulated new urban employment in five years (ten thousand)	A12
		Urban registered unemployment rate (%)	A13
	Social Security (A2)	Pension insurance coverage rate for urban residents (%)	A21
		Urban residents’ medical insurance coverage rate (%)	A22
		Number of elderly care beds per thousand elderly	A23
	Education (A3)	Average years of education of the main workingage population (15-64 years old)	A31
		Average years of education of newly added labor force	A32
		Gross enrollment rate in the three years before school (%)	A33
		Compulsory education completion rate (%)	A34
		Gross enrollment rate in high school (%)	A35
		Gross enrollment rate of higher education (%)	A36
	Medical hygiene (A4)	Number of beds per thousand people	A41
		Number of practicing (assistant) physicians per thousand people	A42
		Maternal mortality rate (one in 100,000)	A43
		Infant mortality rate (1 in 1,000)	A44
	Population and public travel (A5)	Natural population growth rate (one thousandth)	A51
		Life expectancy	A52
		Resident travel public transportation share rate (%)	A53
		Rail transit operating mileage (km)	A54
	Pollution control (A6)	The ratio of days with good air quality in the main urban area (%)	A61
		Harmless treatment rate of urban domestic garbage (%)	A62
		Urban sewage treatment rate (%)	A63
		Water quality compliance rate of drinking water source (%)	A64
	Ecological greening (A7)	Per capita public green area (m)	A71
		Forest cover rate (%)	A72
		Urban greening rate (%)	A73

#### Cluster Analysis Model of Social Development Level of 35 Large and Medium Cities

Cluster analysis is a method of reducing the number of research objects by grouping things with similar properties into one category based on the characteristics of the research objects (samples or indicators). The main principle of the cluster analysis method is to first treat all n samples as different n classes, then merge the two classes with the closest nature (that is, the closest distance) into one class, then find the closest one from the n-1 classes the two categories are merged, and so on until all the samples are merged into one category. The detailed steps are as follows (Murray and Reiter, 2015; Resche-Rigon and White, 2016; Audigier and Niang, 2020):

Step 1: select the analysis variable, and set the original observation data matrix as:


(7) $$X\,\left[\begin{array}{ccc} x_{11} & \cdots & x_{1\,p}\\ \vdots & \ddots & \vdots \\ x_{n\,1} & \cdots & x_{n\,p} \end{array}\right]$$

Step 2: Data standardization. The standardized formula is:


(8) $$x_{i\,j}^{*}=\frac{x_{i\,j}-{\bar{x}}_{j}}{S_{i}}\;\left(i=1,2,3\cdots n;j=1,2,3\cdots p\right)$$

(9) $$\bar{x}=\frac{1}{n}\sum_{i=1}^{n}x_{ij}$$

(10) $$S_{i}=\frac{1}{n-1}\sum_{i=1,j=1}^{n}{\left(x_{i}-\bar{x}\right)}^{2}$$

Step 3: Calculate the new distance $d_{i\,j}^{2}$ between all the data pairs, and generate a distance matrix. Euclidean distance square sum calculation method:


(11) $$d_{ij}^{2}=\sum_{k-1}^{p}{\left(x_{ik}-x_{jk}\right)}^{2}\left(i=1,2,3\cdots n;j=1,2,3\cdots p\right)$$

Step 4: In the choice of clustering method, select the average distance connection method between classes:


(12) $$D_{pq}^{2}=\frac{1}{n_{p}n_{q}}\sum_{x_{i\in G_{p}}}\sum_{x_{j\in G_{p}}}d_{ij}^{2}\left(i=1,2,3\cdots n;j=1,2,3\cdots p\right)$$

Assuming that the clustering reaches a certain step, the class *G_p_* and the class G_q_ are merged into a new classG_r_, then the distance between any class G_k_ and G_r_ is:


(13) $$D_{r\,k}^{2}=\frac{n_{p}\,D_{k\,p}^{2}+n_{q}\,D_{k\,p}^{2}}{n_{p}+n_{q}}$$

Step 5: Continue with the third and fourth steps until all the samples are classified into one category. Finally, according to the background knowledge of social development, according to a certain classification standard or classification principle, the final classification result is obtained.

## Results

### Analysis of the Evaluation Index System of Social Development Level of 35 Large and Medium Cities

**FIGURE 1:**
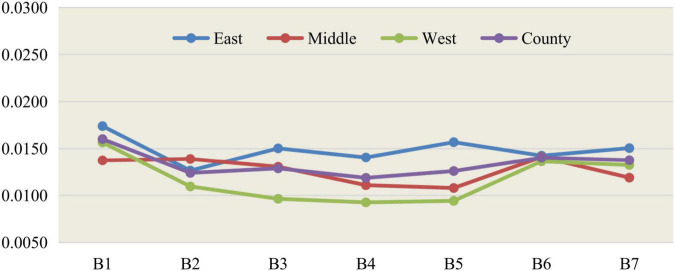
Line chart of social development level scores of three regions from 2011 to 2015.

**FIGURE 2:**
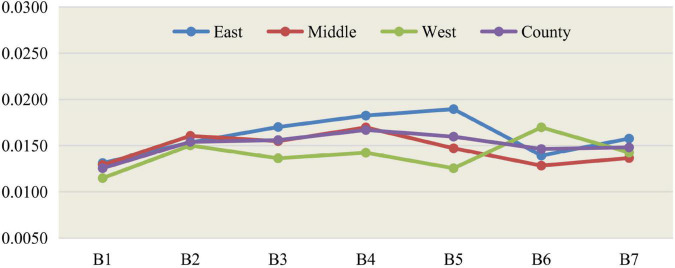
Line chart of social development level scores of three regions from 2016 to 2020.

According to the weights of the second-level indicators of the entropy method of social development of 35 large and medium-sized cities, calculate the scores and total scores of the 35 large and medium-sized cities in 2011–2015 and 2016–2020. The average scores of 2011–2015 and 2016–2020 are used as the comparison object, and the scores of 35 large and medium-sized cities and eastern, central, and western regions are ranked. The analysis results are shown in Tables 2, 3; the related results are drawn as line graphs as shown in Figures 1, 2.

**TABLE 2:** 2011–2015 social development level scores of the three regions.

Region	B1		B2		B3		B4		B5		B6		B7		Total score	Overall ranking
	Score	Rank	Score	Rank	Score	Rank	Score	Rank	Score	Rank	Score	Rank	Score	Rank		
East	0.0174	1	0.0126	2	0.0150	1	0.0140	1	0.0157	1	0.0142	1	0.0150	1	0.1041	1
Middle	0.0137	3	0.0139	1	0.0131	2	0.0111	2	0.0108	2	0.0141	2	0.0119	3	0.0885	2
West	0.0157	2	0.0110	3	0.0096	3	0.0093	3	0.0094	3	0.0136	3	0.0132	2	0.0818	3
County	0.0160		0.0124		0.0129		0.0119		0.0126		0.0140		0.0138		0.0935	

**TABLE 3:** 2016–2020 social development level scores of the three regions.

Region	B1		B2		B3		B4		B5		B6		B7		Total score	Overall ranking
	Score	Rank	Score	Rank	Score	Rank	Score	Rank	Score	Rank	Score	Rank	Score	Rank		
East	0.0131	1	0.0154	2	0.0170	1	0.0183	1	0.0190	1	0.0139	2	0.0158	1	0.1122	1
Middle	0.0129	2	0.0161	1	0.0155	2	0.0170	2	0.0147	2	0.0128	3	0.0137	3	0.1039	2
West	0.0115	3	0.0150	3	0.0136	3	0.0142	3	0.0126	3	0.0170	1	0.0143	2	0.0990	3
County	0.0126		0.0154		0.0156		0.0167		0.0160		0.0146		0.0148		0.1062	

There are obvious differences in the level of social development in the development plans of 35 large and medium cities as shown in the above graphs. During 2011–2015, it can be seen that from the perspective of China’s three major regions, in terms of the scores of the seven major first-level indicators, the eastern part ranked first in employment, education, medical care, population and travel, pollution control, and ecological greening, and only ranked second in social security indicators. At the same time, the western region ranked first in all seven first-level indicators except ecological greening and employment indicators. In addition to the second, they are all located in the third. The total scores of eastern cities, central cities, western cities, and the national level were 0.1041, 0.0885, 0.0818, and 0.0935, respectively. The scores of central and western cities did not reach the average level of 35 large and medium-sized cities and were only 85.01% and 85.01% of those of eastern cities. 78.58%, and the score of western cities is 92.43% of that of central cities.

During 2016–2020, from the perspective of China’s three major regions, in terms of the scores of the seven major first-level indicators, the eastern part is equally well-known in the five first-level indicators of employment, education, medical care, population and travel, and ecological greening. Ranked first, compared with the 2011–2015 period, the first-level pollution control index has changed from first to second, and the western region has changed from third to first in the first-level pollution control index. The central city surpassed the western city in the second place in the employment index. The total scores of eastern cities, central cities, western cities, and the national level were 0.1122, 0.1039, 0.0990, and 0.1062, respectively. The scores of central and western cities did not reach the average level of 35 large and medium-sized cities and were 92.60% and 88.24 of eastern cities. % and the score of western cities is 95.28% of that of central cities.

From a national perspective, from 2011–2015 to 2016–2020, the social development level of 35 large and medium-sized cities across the country has been improved to a certain extent, and the social development gap between cities has also been reduced to a certain extent. From a regional perspective, from the “Twelfth Five-Year” development plan to the “Thirteenth Five-Year” development plan, the social development gap between central cities, western cities, and eastern cities is narrowing, while the social development gap between western and eastern cities is narrowing. The gap is also narrowing.

### Cluster Analysis of Social Development Levels of 35 Large and Medium Cities

Through the cluster analysis of the collected raw data through SPSS software, the cluster analysis pedigree of the social development of 35 large and medium-sized cities in the 2011–2015 period and the 2016–2020 period can be obtained Based on the pedigree diagram principle, the closer ones are merged into one category, and the distance in the tree diagram is ten as the standard. It can be seen that the pedigree diagrams of 35 large and medium-sized cities during the 2011–2015 social development cluster analysis are classified into four categories. It is reasonable to divide the cluster analysis pedigree diagram of the social development of 35 large and medium-sized cities into three categories during the 2016–2020 period. See Tables 4, 5 for the specific classification.

**TABLE 4:** Classification and summary of the social development of 35 large and medium-sized cities in the 2011–2015 period.

Category	City
The first category	Beijing, Shanghai, Guangzhou
Second category	Tianjin, Chongqing, Shenzhen
Third category	Changsha, Xi’an, Shenyang, Wuhan, Chengdu, Dalian, Nanjing, Hangzhou
Fourth category	Haikou, Hohhot, Lanzhou, Urumqi, Yinchuan, Guiyang, Kunming, Shijiazhuang, Taiyuan, Hefei, Jinan, Changchun, Nanchang, Harbin, Nanning, Qingdao, Xiamen, Fuzhou, Zhengzhou, Ningbo, Xining

**TABLE 5:** Classification and summary of the social development of 35 large and medium-sized cities in the 2016–2020 period.

Category	City
The first category	Chengdu
Second category	Beijing, Chongqing, Shenzhen, Shanghai, Tianjin, Xiamen, Guangzhou, Kunming, Hangzhou, Qingdao, Nanjing, Wuhan, Changsha, Xi’an, Dalian, Jinan
Third category	Changchun, Yinchuan, Haikou, Xining, Harbin, Hefei, Zhengzhou, Shenyang, Fuzhou, Ningbo, Nanchang, Guiyang, Hohhot, Lanzhou, Urumqi, Taiyuan, Nanning, Shijiazhuang

It can be seen from Tables 4, 5 that during 2011–2015, 35 large and medium-sized cities were divided into four categories, and during 2016–2020, they were reduced to three categories, which, to a certain extent, explained that in the past decade, the social development gap between China’s 35 large and medium-sized cities has narrowed. Specifically, during 2011–2015, the number of cities in the first and second categories was three, namely Beijing, Shanghai, Guangzhou, Tianjin, Chongqing, and Shenzhen, and the number of cities in the fourth category reached 21, and during 2016–2020, only Chengdu is in the first category city standard, and the number of other second and third category cities is not much different. This shows that in the past decade, with the rapid development of various large and medium-sized cities, the comprehensive level of urban social development has been continuously improved, and the gap between cities has been narrowing.

## Discussion

### The Social Development Gap Between China’s 35 Large and Medium Cities

According to the comprehensive scores of the social development of 35 large and medium-sized cities obtained from previous calculations, Table 6 can be obtained by collating and calculating.

**TABLE 6:** The social development gap between China’s 35 large and medium cities.

Period	Overall ratings	Standard deviation	Coefficient of variation	The ratio of the highest to the lowest (times)
2011–2015	0.0935	0.0167	0.1786	2.0865
2016–2020	0.1062	0.0158	0.1488	1.9433

The data shows that compared with the 2011–2015 period, the social development of the 2016–2020 period has significantly improved. The coefficient of variation obtained by dividing the standard deviation by the comprehensive score of social development also dropped significantly. In addition, even if a partial comparison index such as “the ratio of the highest to the lowest” is used to measure the social development gap between 35 large and medium-sized cities during 2011–2015 and 2016–2020, the data also shows that this gap has been obtained. Decrease slightly. Therefore, we can conclude that compared with 2011–2015, the social development gap between 35 large and medium-sized cities in China has narrowed during 2016–2020.

### The Social Development Gap Between the Three Major Regions in the East, Middle, and West

It can be seen from Table 7 that, from the perspective of three major regions, the gap in the level of social development among the three major regions of the east, middle, and west has declined from the 2011–2015 period to the 2016–2020 period. From the comparison results of the three major regions of the east, the middle and the west, the social development gaps between the east and the west, between the east and the middle, and between the middle and the west are all shrinking. The gap in the development of China has narrowed the most, at 10.83%.

**TABLE 7:** The social development gap in China ’s eastern, middle, and western regions.

Period	Standard deviation	Coefficient of variation	The ratio of east to west (take west as 1)	The ratio of eastern to middle (take middle as 1)	The ratio of the central part to the western part (take west as 1)
2011–2015	0.0093	0.0999	1.2726	1.1763	1.0819
2016–2020	0.0054	0.0513	1.1333	1.0799	1.0495

### The Social Development Gap Between the Eastern, Central, and Western Regions

Based on the social development part of 35 large and medium-sized city development plans, this paper has obtained the regional comprehensive scores of 17 urban agglomerations during the 2011–2015 and 2016–2020 periods to analyze the areas with the social development level of the three major regions. This manuscript divides the 35 large and medium-sized cities according to the urban agglomerations into the eastern, central, and western regions and elaborates on the gaps in the social development level of these regions.

#### Disparities in Social Development Within the Eastern Region

**FIGURE 3:**
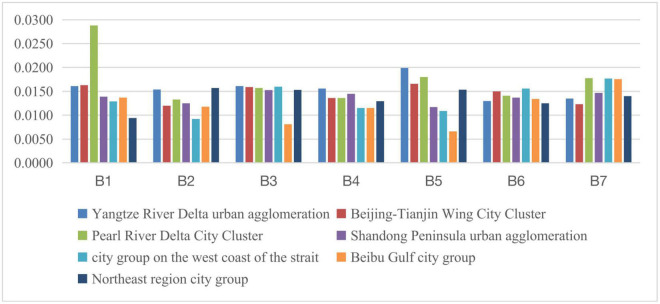
Social development level of each indicator within the eastern region during 2011–2015.

**FIGURE 4:**
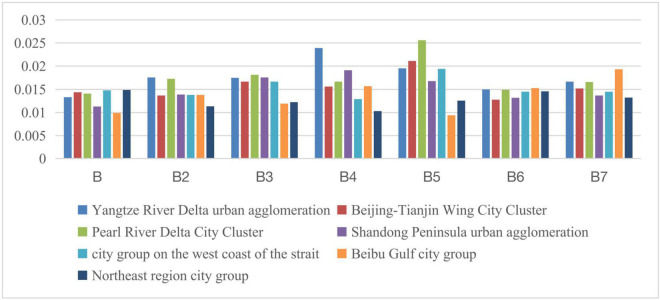
Social development level of each indicator in the eastern region during 2016–2020.

The eastern region is mainly comprised of seven regions, namely, the Beijing-Tianjin Wing City Cluster, the Yangtze River Delta urban agglomeration (Shanghai, Nanjing, Hangzhou, Ningbo), the Pearl River Delta City Cluster (Guangzhou, Shenzhen), Shandong Peninsula urban agglomeration (Ji’nan, Qingdao), city group on the west bank of the Straits (Fuzhou, Xiamen), Beibu Gulf city group (Nanning, Haikou), and Northeast region city group (Harbin, Changchun, Jilin). The social development level of each indicator within the eastern region during 2011–2015 is shown in Figures 3, 4. Indicators related to the gap in social development levels among urban agglomerations in eastern China can be seen in Table 8.

**TABLE 8:** Indicators related to the gap in social development level among urban agglomerations in eastern China.

Statistical indicators	2011–2015	2016–2020
Standard deviation	0.0121	0.0102
Coefficient of variation	0.1160	0.0907
The ratio of the Yangtze River Delta to the Pearl River Delta (take the Pearl River Delta as 1)	0.9050	1.0008
The ratio between the Yangtze River Delta and Beijing-Tianjin-Hebei (take Beijing-Tianjin-Hebei as 1)	1.0777	1.1354
The ratio of the Yangtze River Delta to the Shandong Peninsula (take the Shandong Peninsula as 1)	1.1417	1.1687
The ratio of the Yangtze River Delta to the west coast of the Strait (take the west coast of the Strait as 1)	1.1722	1.1588
The ratio of the Yangtze River Delta to the Beibu Gulf urban agglomeration (take the Beibu Gulf urban agglomeration as 1)	1.3317	1.2958
The ratio of the Yangtze River Delta to the Northeast (take the Northeast as 1)	1.2273	1.2505
The ratio between the Pearl River Delta and Beijing-Tianjin-Hebei (take Beijing-Tianjin-Hebei as 1)	1.1908	1.1344
The ratio of the Pearl River Delta to the Shandong Peninsula (take the Shandong Peninsula as 1)	1.2615	1.1678
The ratio of the Pearl River Delta to the west coast of the Strait (take the west coast of the Strait as 1)	1.2952	1.1579
The ratio of the Pearl River Delta to the Beibu Gulf City Group (take the Beibu Gulf City Group as 1)	1.4714	1.2948
The ratio of the Pearl River Delta to the Northeast (take the Northeast as 1)	1.3561	1.2495
The ratio of Beijing-Tianjin-Hebei to Shandong Peninsula (take Shandong Peninsula as 1)	1.0594	1.0294
The ratio of Beijing-Tianjin-Hebei to the west coast of the Strait (take the west coast of the Strait as 1)	1.0877	1.0207
The ratio of Beijing-Tianjin-Hebei and Beibu Gulf urban agglomeration (take the Beibu Gulf urban agglomeration as 1)	1.2357	1.1414
The ratio of Beijing-Tianjin-Hebei to the Northeast (take the Northeast as 1)	1.1389	1.1014
The ratio of Shandong Peninsula to the west coast of the Strait (take the west coast of the Strait as 1)	1.0267	0.9915
The ratio of Shandong Peninsula to Beibu Gulf city group (take Beibu Gulf city group as 1)	1.1665	1.1088
The ratio of Shandong Peninsula to the Northeast (take the Northeast as 1)	1.0750	1.0700
The ratio of the west bank of the Strait to the Beibu Gulf city group (take the Beibu Gulf city group as 1)	1.1361	1.1182
The ratio of the west coast of the Straits to the northeast (take the northeast as 1)	1.0470	1.0791
The ratio of Beibu Gulf urban agglomeration to the ratio of Northeast China (take Northeast China as 1)	0.9216	0.9650

It can be seen from Table 8 that, as a whole, the internal social development gap in the eastern region is showing a downward trend, but the situation is different for each urban agglomeration. The Yangtze River Delta urban agglomeration is relative to the Pearl River Delta, Beijing-Tianjin-Hebei, Shandong Peninsula, and Northeast China. The social development gaps are all showing an expanding trend. The Pearl River Delta urban agglomeration, the Beijing-Tianjin-Hebei urban agglomeration, and the Shandong Peninsula urban agglomeration have narrowed their social development gaps relative to other urban agglomerations in the eastern region. The social development of the urban agglomerations on the west bank of the Straits and Beibu Gulf is relative to the social development of the Northeast. The gap has widened. Therefore, we can think that the reason for the narrowing of the overall social development gap in the eastern region is due to the slower social development of the Beijing-Tianjin-Hebei urban agglomeration, the Shandong Peninsula urban agglomeration, the northeastern region, and the Pearl River Delta urban agglomeration relative to other urban agglomerations.

#### Disparities in Social Development Within the Middle Region

**FIGURE 5:**
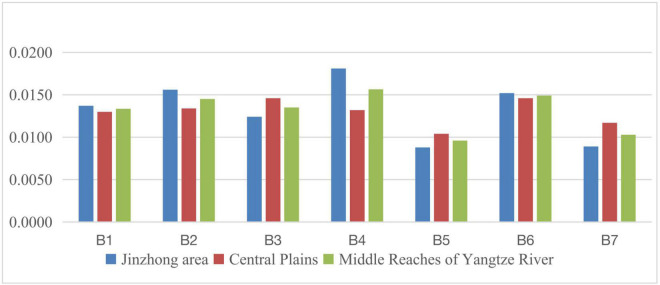
Social development level of each indicator within the middle region during 2011–2015.

**FIGURE 6:**
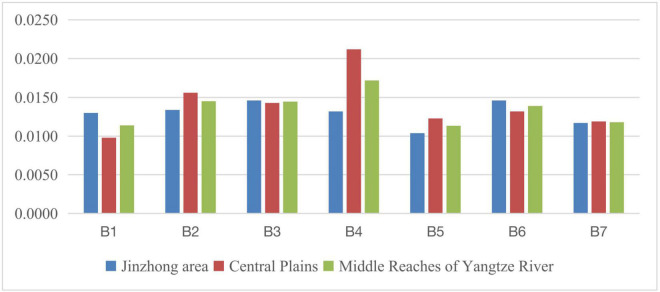
The social development level of each indicator in the middle region during 2016–2020.

The middle region is mainly comprised of the Jinzhong area (Taiyuan), Central Plains (Zhengzhou), and the Middle Reaches of the Yangtze River (Wuhan, Changsha, Nanchang, and Hefei). By calculating the relevant index data, the social development levels of each indicator within the middle region during 2011–2015 and 2016–2020 are shown in Figures 5, 6. The relevant indicators of the social development level gap between the urban agglomerations in the central region are shown in Table 9.

**TABLE 9:** Indicators related to the gap in social development level among urban agglomerations in middle China.

Statistical indicators	2011–2015	2016–2020
Standard deviation	0.0027	0.0037
Coefficient of variation	0.0310	0.0355
The ratio of the Jinzhong area to the Central Plains area (take the Central Plains as 1)	1.0220	0.9398
The ratio of Jinzhong area to the city group in the middle reaches of the Yangtze River (take the city group in the middle reaches of the Yangtze River as 1)	1.0759	0.9183
The ratio of the Central Plains area to the urban agglomeration in the middle reaches of the Yangtze River (take the urban agglomeration in the middle reaches of the Yangtze River as 1)	1.0528	0.9771

According to the graphs, on the whole, from 2011–2015 to 2016–2020, the social development gap between the central regions has shown an upward trend, mainly reflected in the rapid development of the Central Plains and the middle reaches of the Yangtze River; especially, the middle reaches of the Yangtze River was still at the end of the three major regions during the 2011–2015 period and has risen to the top of the three major regions during the 2016–2020 period.

#### The Social Development Gap Within the Western Region

**FIGURE 7:**
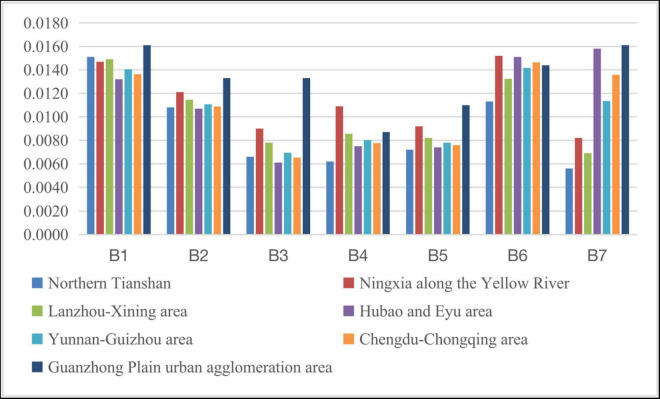
Social development level of each indicator within the western region during 2011–2015.

**FIGURE 8:**
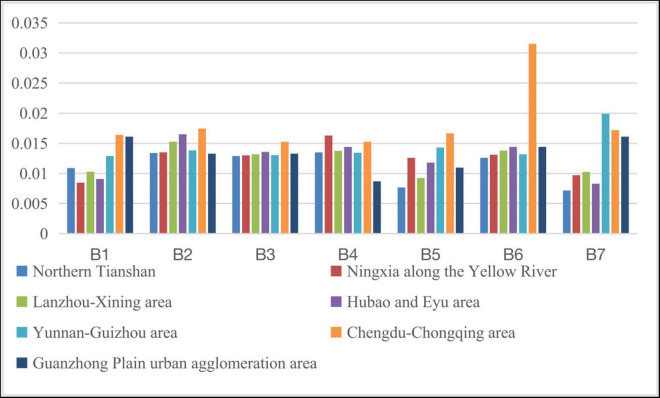
Social development level of each indicator within the western region during 2016–2020.

The western region mainly comprises Northern Tianshan (Urumqi), Ningxia along the Yellow River (Yinchuan), Lanzhou-Xining area, Hubao and Eyu area (Hohhot), Yunnan-Guizhou area (Kunming, Guiyang), Chengdu-Chongqing area, and Guanzhong Plain urban agglomeration area (Xi’an). By calculating the relevant index data, the social development levels of each indicator within the middle region during 2011–2015 and 2016–2020 are shown in Figures 7, 8. The relevant indicators of the social development level gap between the urban agglomerations in the central region are shown in Table 10.

**TABLE 10:** Relevant indicators of the social development gap between urban agglomerations in western China.

Statistical indicators	2011–2015 Period	2016–2020 Period
Standard deviation	0.0132	0.0160
Coefficient of variation	0.1612	0.1615
The ratio of Chengyu area to Guanzhong Plain area (take Guanzhong Plain area as 1)	1.1421	1.2035
The ratio of Chengdu-Chongqing area to Yun-Gui area (take Yun-Gui area as 1)	1.3664	1.2873
The ratio of Chengyu area to HubaoEyu area (take HubaoEyu area as 1)	1.4016	1.4699
The ratio of hengdu-Chongqing area to Lanzhou-Xining area (with Lanzhou-Xining area being 1)	1.4367	1.4583
The ratio of Chengdu-Chongqing area to the area along the Yellow River in Ningxia (take the area along the Yellow River in Ningxia as 1)	1.3887	1.4954
The ratio of Chengdu-Chongqing area to the northern part of Tianshan (take the northern part of Tianshan as 1)	1.6922	1.6560
The ratio of Guanzhong plain area to Yungui area (take Yungui area as 1)	1.1964	1.0696
The ratio of Guanzhong plain area to HubaoEyu area (take HubaoEyu area as 1)	1.2272	1.2213
The ratio of the Guanzhong Plain area to the Lanzhou-Xining area (with the Lanzhou-Xining area being 1)	1.2580	1.2117
The ratio of the Guanzhong plain area to the area along the Yellow River in Ningxia (take the area along the Yellow River in Ningxia as 1)	1.2160	1.2425
The ratio of the Guanzhong plain area to the northern Tianshan area (take the northern Tianshan area as 1)	1.4817	1.3760
The ratio of Yunnan-Guizhou area to HubaoEyu area (take HubaoEyu area as 1)	1.0258	1.1419
The ratio of Yunnan-Guizhou area to Lanzhou-Xining area (with Lanzhou-Xining area being 1)	1.0515	1.1329
The ratio of Yunnan-Guizhou area to the area along the Yellow River in Ningxia (take the area along the Yellow River in Ningxia as 1)	1.0164	1.1617
The ratio of Yunnan-Guizhou area to the northern part of Tianshan (take the northern part of Tianshan as 1)	1.2384	1.2864
The ratio of HubaoEyu area to Lanzhou-Xining area (with Lanzhou-Xining area being 1)	1.0251	0.9921
The ratio of HubaoEyu area to the area along the Yellow River in Ningxia (take the area along the Yellow River in Ningxia as 1)	0.9908	1.0173
The ratio of the HubaoEyu area to the northern Tianshan area (take the northern Tianshan area as 1)	1.2073	1.1266
The ratio of Lanzhou-Xining area to the area along the Yellow River in Ningxia (take the area along the Yellow River in Ningxia as 1)	0.9666	1.0254
The ratio of Lanzhou-Xining area to the northern part of Tianshan (take the northern part of Tianshan as 1)	1.1778	1.1355
The ratio of the area along the Yellow River in Ningxia to the northern part of Tianshan (take the northern part of Tianshan as 1)	1.2185	1.1074

According to the graphs, the social development gap in the western region shows an overall expansion trend. However, due to the large difference in the absolute value of the social development level between the various regions in the western region, the coefficient of variation shows that the social development gap in the western region has not increased significantly. Specifically, compared with the Guanzhong Plain, HubaoEyu, Lanzhou-Xining, and Ningxia along the Yellow River, the gap in social development in the Chengdu-Chongqing area is expanding; the Guanzhong Plain is only relative to the level of social development in Ningxia along the Yellow River. Compared with other regions, the social development level of the Yunnan-Guizhou region has risen significantly. The HubaoEyu region has increased compared to the Yellow River region in Ningxia and the northern Tianshan region. The Lanzhou-Xining region has risen significantly compared to the area along the Yellow River in Ningxia. In short, among the various regions within the western region, from 2011–2015 to 2016–2020, the level of social development in the Chengdu-Chongqing region, the Yunnan-Guizhou region, and the northern part of the Tianshan Mountains has increased significantly. The relative level of development has dropped significantly.

## Conclusion

Existing literature studies on regional development gaps mostly focus on the microeconomic scale based on economic statistics (GDP) or social survey data and rarely pay attention to the impact of government policies on regional development gaps from the perspective of government macro-social development. In order to reduce this deficiency, we use the 2011–2015 annual development plan, the 2016–2020 annual development plan, and the special development plan data of each city to characterize the urban development gap and construct a comprehensive evaluation model for the social development of 35 large and medium-sized cities in China for research and discussion on the social development gap of 35 large and medium cities in China, the trend of social development gap in the eastern, central, and western regions and the social development gap in the eastern, central, and western regions. The research results show that:

1. Looking at the whole country, there are obvious differences in the social development level of the 35 large and medium-sized cities in the development plan, but from 2011–2015 to 2016–2020, the social development level of the 35 large and medium-sized cities in the country has achieved a certain level of development. As a result, the social development gap between cities has also been reduced to a certain extent. During 2011–2015, the highest score of 35 large and medium-sized cities was 0.13099 in Shenzhen, the lowest was 0.06278 in Urumqi, and the national average score was 0.0935. The 35 large and medium-sized cities have all achieved social development from 2011 to 2015. There are 13 cities whose level is higher than the national average. During 2016–2020, the highest score of 35 large and medium-sized cities was 0.15193 points in Chengdu, the lowest score was 0.07818 points in Urumqi, and the national average score was 0.1062 points, of which 35 large and medium-sized cities in the period 2016–2020. There are 17 cities whose social development level is higher than the national average.
2. From 2011–2015 to 2016–2020, the gap in social development levels among the three major regions of China’s eastern, central, and western regions has decreased. Judging from the pairwise comparison results of the three major regions of the east, the middle, and the west, the social development gap between the east and the west, between the east and the middle, and between the middle and the west is narrowing. The development gap narrowed the most, at 10.83%.
3. The trend of social development disparity within the three major regions of the eastern, central, and western regions is not the same. The internal social development gap in the eastern region shows a downward trend, while the internal social development gap in the central and western regions shows an upward trend. From 2011–2015 to 2016–2020, within the three major regions of the East and Midwest. With the rapid development of the social development level of the Yangtze River Delta urban agglomeration, the urban agglomeration in the middle reaches of the Yangtze River, and the Chengdu-Chongqing urban agglomeration, accompanied by the relatively slow social development speed of the Beijing-Tianjin-Hebei, Northeast, Jinzhong, and Guanzhong Plain regions, it has become the driving force for all regions in my country. The main reason for the level of social development in the unit. At the same time, compared with the decline in the social development gap between the three original eastern, central, and western regions, the social development gap between the northern and southern regions of China is constantly expanding.

Cities are areas of intensive economic and social development. Coordinated development between cities is conducive to a more frequent flow of resources and factors, more convenient cross-regional development of enterprises, and closer economic and trade links and social exchanges between cities, which can do good to the formation of the internal Contact Network. Further, the development of the connection network between cities will play a guiding role in the flow of factors and the cross-regional development of enterprises, promote the optimal allocation of factors, and help jointly solve cross-regional problems in public areas such as transportation, communication, resource development, and environmental protection. Reduce transaction costs, form functional differentiation with the industrial division of labor and cooperation as the main content, and thus form increasingly close economic ties and development dependencies and interactions.

This manuscript corroborates that in the past decade, China’s regional social development gap has generally narrowed, and China’s economic reforms have not exacerbated regional development imbalances, but have brought economic prosperity and opportunities to all. It provides evidence for scholars to propose continuing the coast-oriented regional development policy. However, we should also see that in the context of the overall decline in the regional social development gap in China, the social development gap within the central and western regions of China has shown an upward trend. This is mainly due to the strong siphon effect from a small number of core cities in the central and western regions to the surrounding cities, attracting a large number of resources from the surrounding cities. In addition, the regional social development in the central and western regions of China was originally backward, resulting in a more prominent problem of the one-way flow of resources from surrounding cities to core cities. In the long run, it has led to a growing disparity in regional social development in the central and western regions of China.

Based on the above research conclusions, we provide the following policy implications: First, show more support for cities in the central and western regions, strengthen the connection between cities in the central and western regions, enhance the advantages of urban agglomerations, and promote the overall development of the region. Second, give full play to the leading and radiating role of central cities and the leading role of advanced core cities in surrounding cities, accelerating the development of underdeveloped areas with concentrated elements in central cities and coordinating regional development. Third, the government’s policies could appropriately favor the central and western regions, increasing the streamlining of administration and delegate power, improving the business environment, and encouraging social forces to enter related fields, actively using the Internet information platform to develop various innovative models such as social security, housing security, education, and medical care.

In this study, we use the data from 2011–2015 to 2016–2020 and the special development plan of each city formulated by the governments of 35 large and medium-sized cities to characterize urban development levels by establishing a comprehensive evaluation model. This research perspective overcomes the limitation and one-sidedness of the original use of economic statistics to measure regional urban development.

## Data Availability Statement

The datasets presented in this study can be found in online repositories. The names of the repository/repositories and accession number(s) can be found in the article/supplementary material.

## Author Contributions

WL and HL identified the research theme and provided meaningful guidance during the whole process. ZL and YW participated in translation and modification. ZL and LW conducted the literature analyzing and collecting and wrote the script. All authors contributed to the article and approved the submitted version.

## Conflict of Interest

The authors declare that the research was conducted in the absence of any commercial or financial relationships that could be construed as a potential conflict of interest.

## Publisher’s Note

All claims expressed in this article are solely those of the authors and do not necessarily represent those of their affiliated organizations, or those of the publisher, the editors and the reviewers. Any product that may be evaluated in this article, or claim that may be made by its manufacturer, is not guaranteed or endorsed by the publisher.
